# Reported characteristics of children referred from primary care to pediatric allergy specialist care

**DOI:** 10.1186/2045-7022-3-S3-P68

**Published:** 2013-07-25

**Authors:** TM Brakel, BMJ Flokstra-de Blok, JNG Oude Elberink, MLA Schuttelaar, WA Christoffers, EM Roerdink, T van der Molen, AEJ Dubois

**Affiliations:** 1 Department of General Practice, University Medical Center Groningen, Groningen, the Netherlands; 2 Department of Allergology, University Medical Center Groningen, Groningen, the Netherlands; 3 Department of Dermatology, University Medical Center Groningen, Groningen, the Netherlands; 4 Department of Pediatric Pulmonology and Pediatric Allergy, University Medical Center Groningen, Groningen, the Netherlands

## Background

For the majority of patients suspected of allergy, management is mostly the responsibility of general practitioners (GPs), but may include specialist referral in selected cases. The purpose of this study is to obtain insight in characteristics of children that are referred from primary care to pediatric specialist care for diagnosis and treatment.

## Methods

Children referred September 2011 and October 2012 to a pediatric allergy clinic were sent a questionnaire which was completed at home. Parents of referred children reported about their child’s allergy.

## Results

Of the questionnaires that were sent out 62% was returned. From the children (*n* = 81, *M*_age_ = 8 years; range 3 months to 17 years) 57% was male. For 17.3% of the children no diagnosis had been established, 1 allergy diagnose was reported in 25.9% of the referred children, and 56.7% reported to have a diagnose for multiple allergies. Of the children for whom one or more diagnoses were reported, 59.8% had eczema, 43.9% food allergy, 29.2% rhinitis, 23.2% asthma and 24.4% had another allergy, not specified. Reference to the specialist due to anaphylactic reactions was reported in 81.6% of the cases. The anaphylactic reactions were caused by food products (80.3%), drugs (9.1%), insect venoms (6.1%), and unknown cause (4.5%). Reported symptoms of anaphylaxis to foods, drugs, and insect venoms were: dizziness and/or palpitations 12.1%, loss of consciousness 6.1%, nausea and/or abdominal cramps 37.9%, vomiting and/or diarrhea 24.2%, itching in the mouth, ears and/or throat 36.4%, itching of the tongue and/or lips 19.7%, itchy and/or watery eyes 37.9%, tongue and/ or lips swelling 28.8%, tightness of the throat 37.9%, and cough 28.8%. It was reported what quantity of food caused complaints, in 64.3% it were crumbs to a few bites /sips, in 12.5% it was a daily portion, and 23.2% did not know. The most common allergenic foods suspected to cause the allergy in food induced anaphylaxis were peanuts 62.3%, milk 34%8%, hazelnut 30.2%, walnut 26.4%, egg 24.5%, cashew 24.5%, pistachio 24.5%, and almond 20.8%.

## Conclusion

Anaphylaxis was the most frequently suspected diagnosis leading to specialist referral and foods were the most frequent cause of anaphylaxis. This suggests that GP’s seek specialist support most often for this category of patients. Our next step will be to evaluate the management plan developed by specialists for these 81 children.

## Disclosure of interest

None declared.

